# Supplementary material for: Surveillance and Genetic Analysis of Low-Pathogenicity Avian Influenza Viruses Isolated from Feces of Wild Birds in Mongolia, 2021 to 2023
Source: Animals (Basel). 2024 Apr 4;14(7):1105. doi: 10.3390/ani14071105 (PMC11011059; doi:10.3390/ani14071105)
Supplement: Supplementary file 1 [file animals-14-01105-s001.zip › Supplemetary figure S1..pptx]

## Slide 1
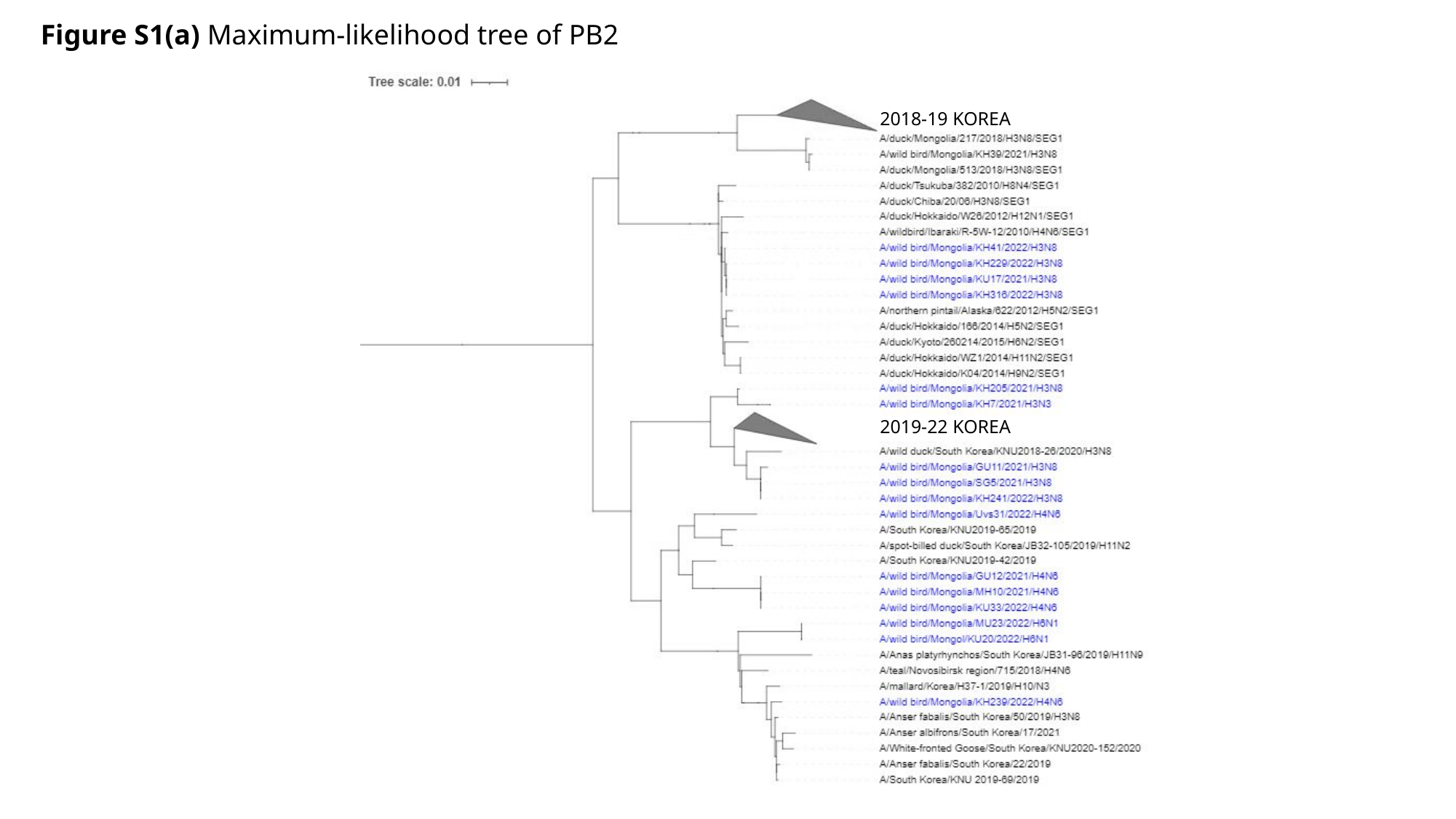

Figure S1(a) Maximum-likelihood tree of PB2
2018-19 KOREA
2019-22 KOREA

## Slide 2
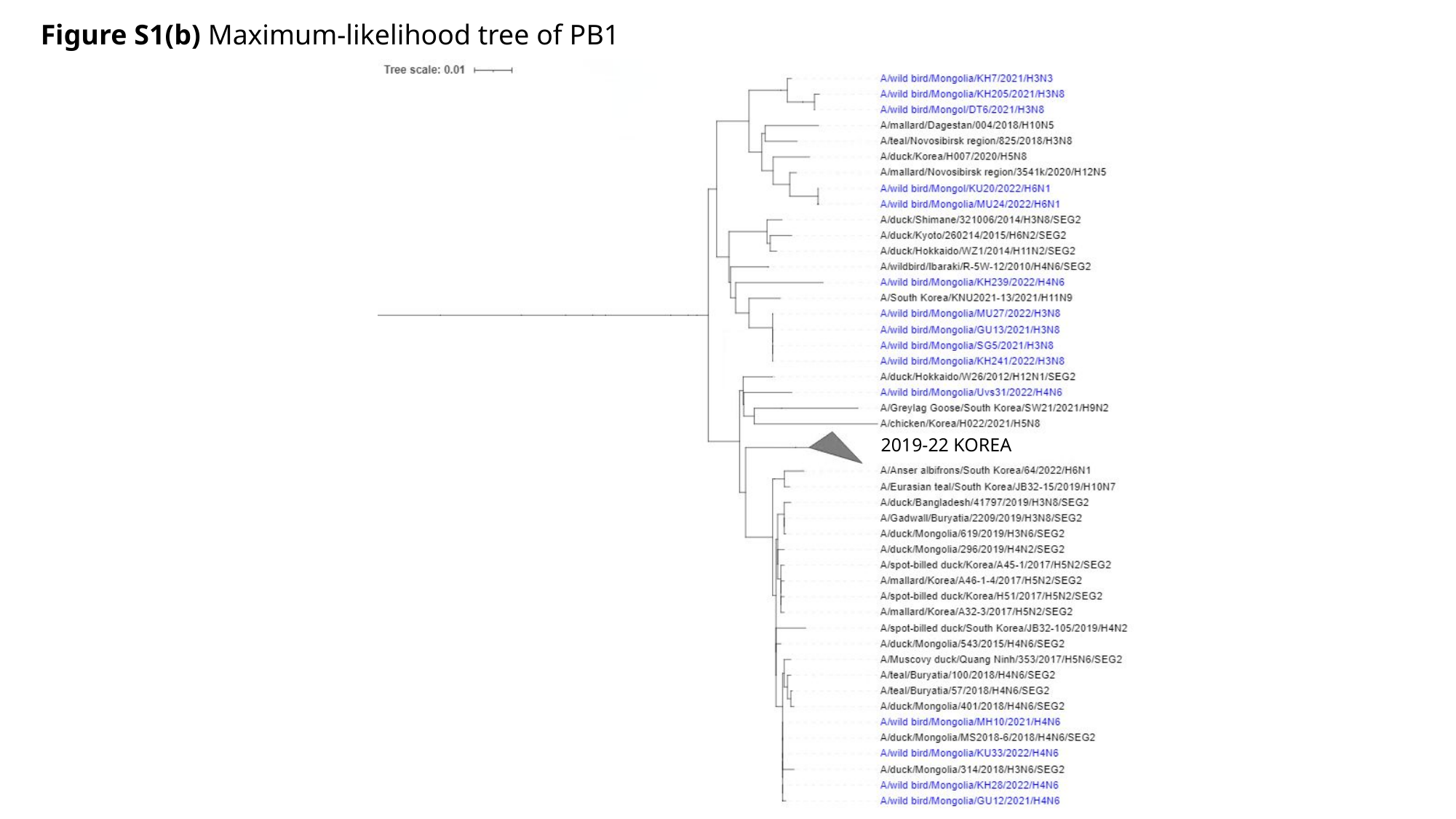

Figure S1(b) Maximum-likelihood tree of PB1
2019-22 KOREA

## Slide 3
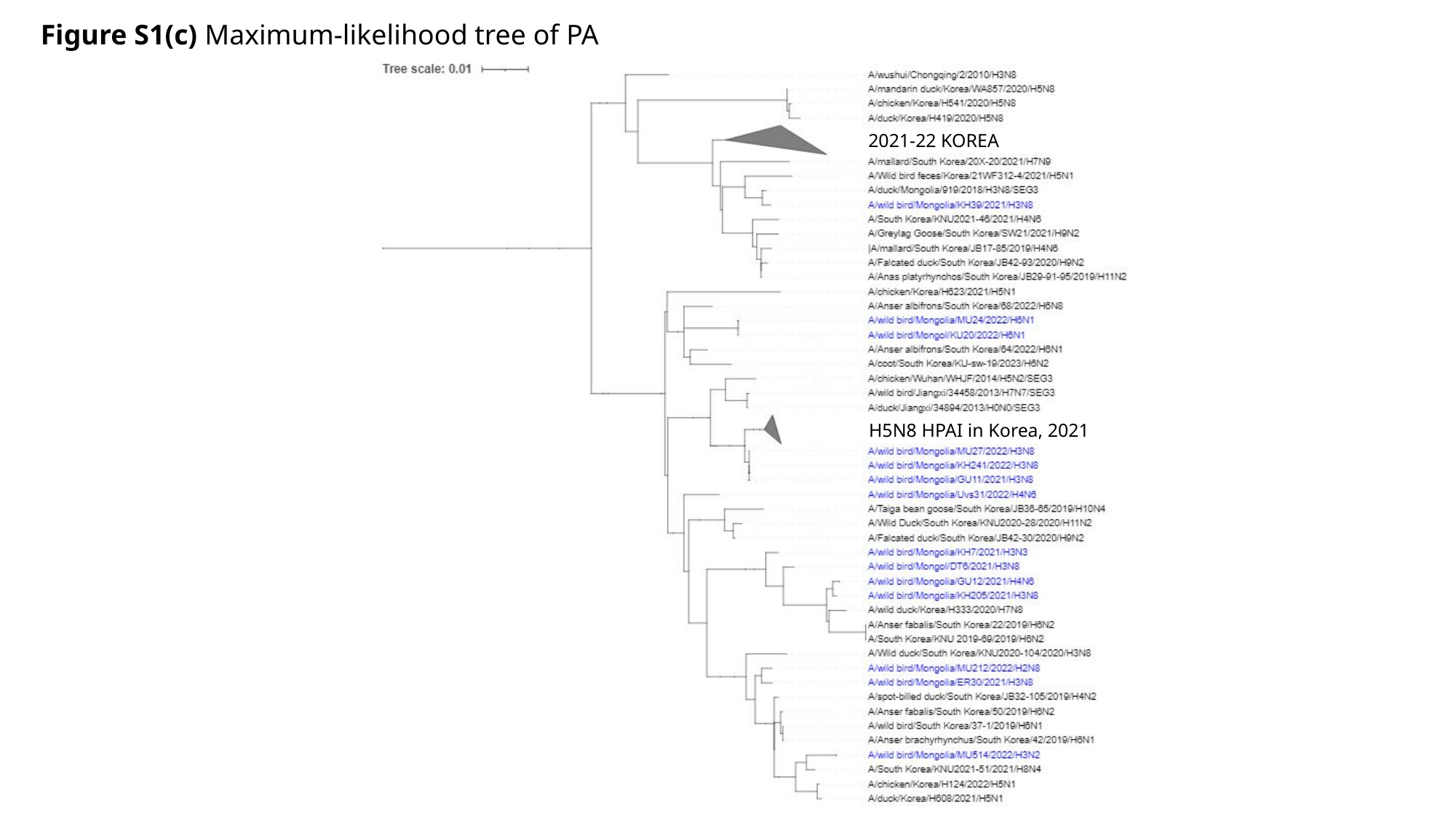

Figure S1(c) Maximum-likelihood tree of PA
2021-22 KOREA
H5N8 HPAI in Korea, 2021

## Slide 4
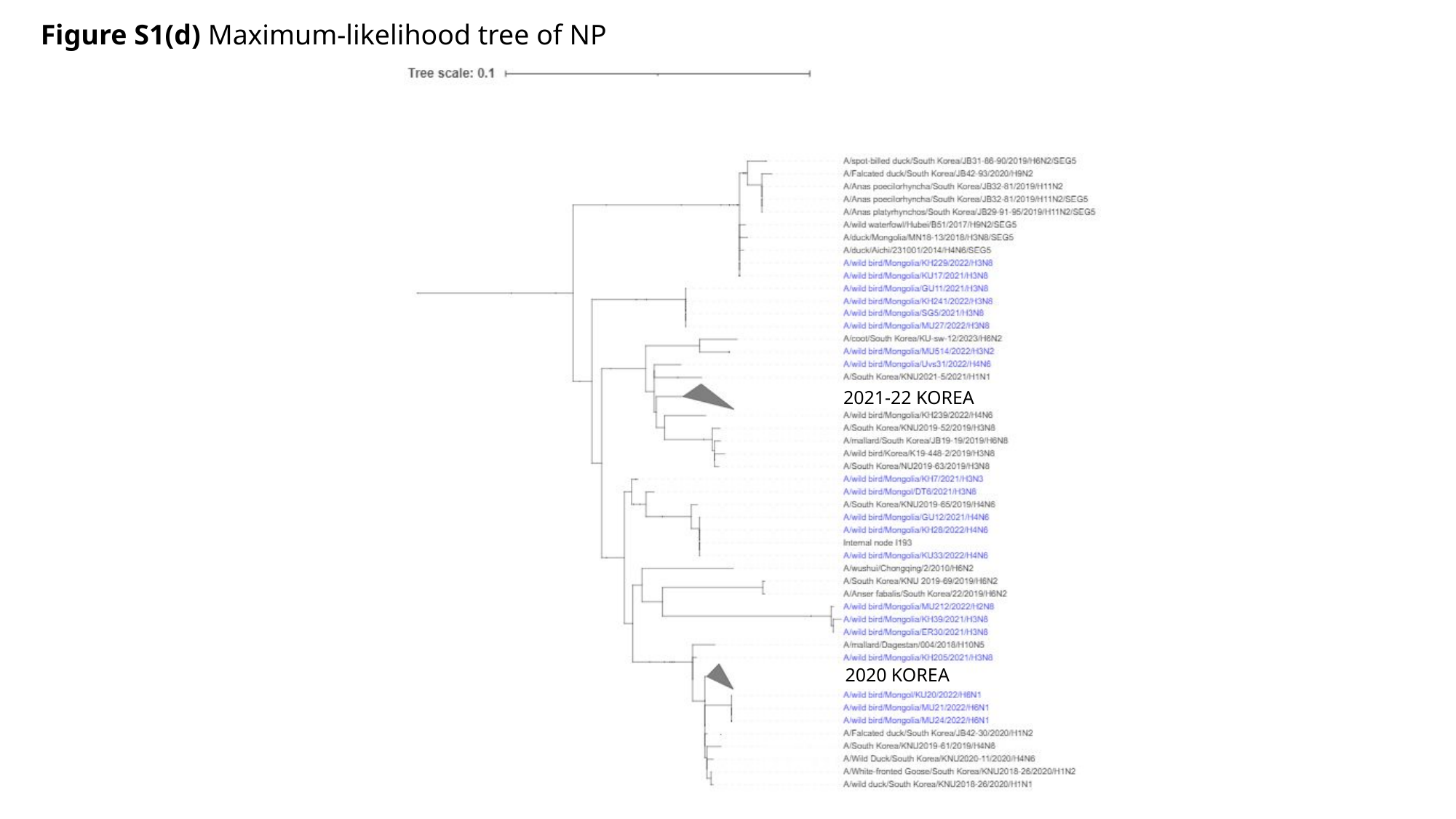

Figure S1(d) Maximum-likelihood tree of NP
2021-22 KOREA
2020 KOREA

## Slide 5
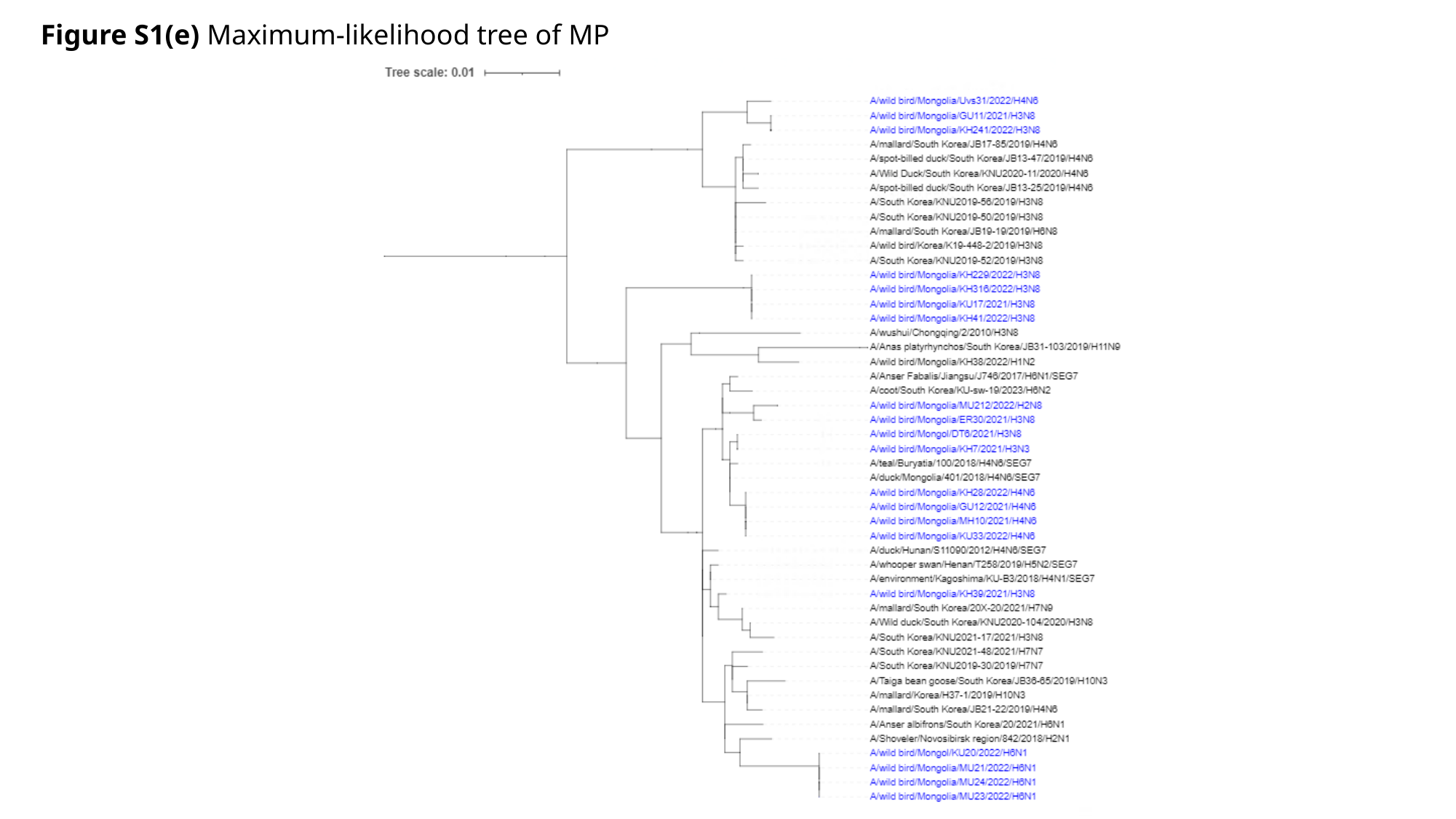

Figure S1(e) Maximum-likelihood tree of MP

## Slide 6
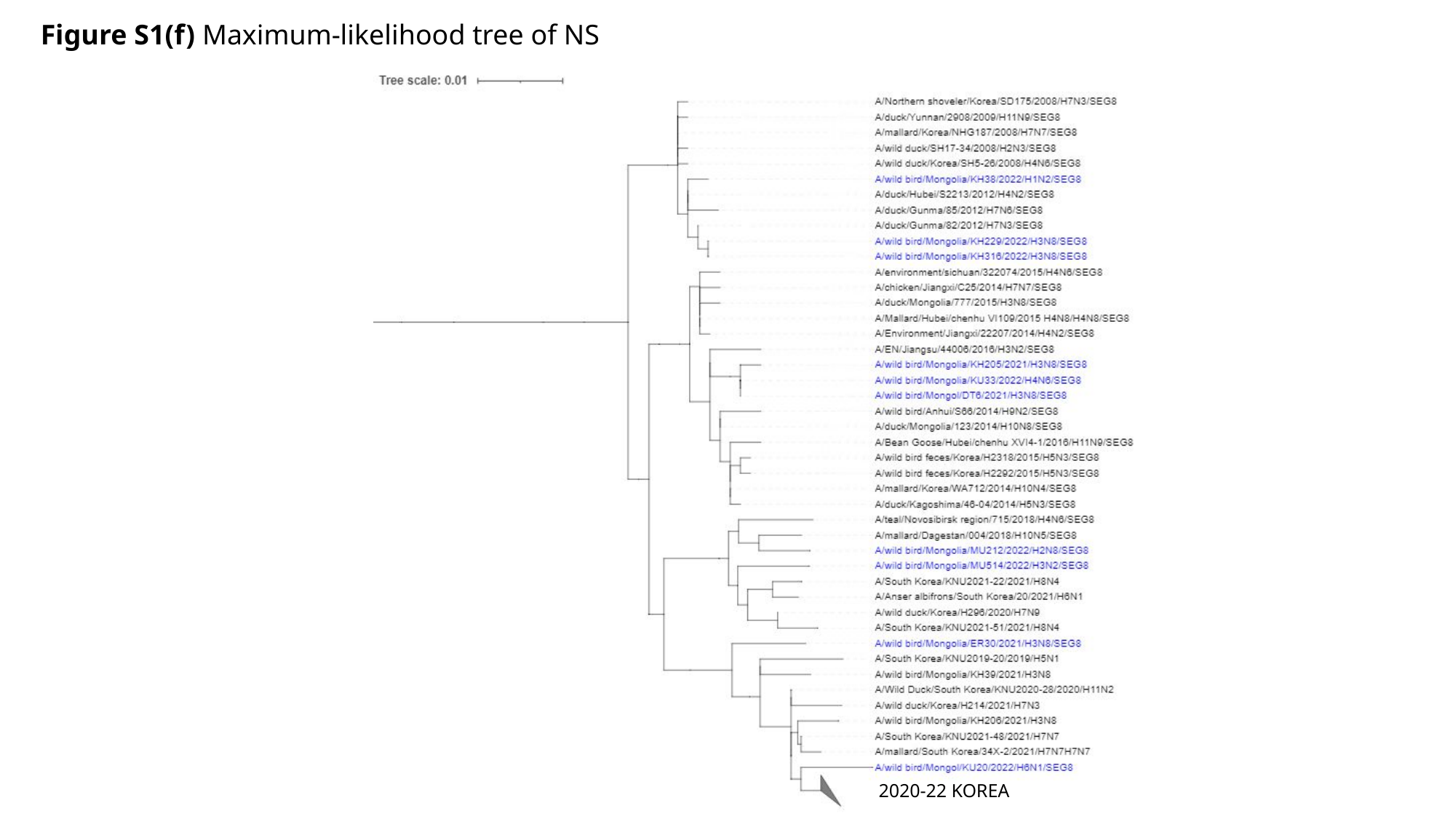

Figure S1(f) Maximum-likelihood tree of NS
2020-22 KOREA
